# The Significance of Chondroitin Sulfate Proteoglycan 4 (CSPG4) in Human Gliomas

**DOI:** 10.3390/ijms19092724

**Published:** 2018-09-12

**Authors:** Davide Schiffer, Marta Mellai, Renzo Boldorini, Ilaria Bisogno, Silvia Grifoni, Cristiano Corona, Luca Bertero, Paola Cassoni, Cristina Casalone, Laura Annovazzi

**Affiliations:** 1 Professor Emeritus of Neurology, University of Turin, Corso Bramante 88/90, 10126 Turin, Italy; davide.schiffer@unito.it; 2 Department of Health Sciences, School of Medicine, University of Eastern Piedmont, 28100 Novara, Italy; martamel73@gmail.com (M.M.); renzo.boldorini@med.uniupo.it (R.B.); 3 Former Research Centre/Policlinico di Monza Foundation, Via P. Micca 29, 13100 Vercelli, Italy; ilaria.bisogno01@universitadipavia.it (I.B.); lannov16@gmail.com (L.A.); 4 Istituto Zooprofilattico Sperimentale del Piemonte, Liguria e Valle d’Aosta, 10126 Turin, Italy; silvia.grifoni@izsto.it (S.G.); cristiano.corona@izsto.it (C.C.); 5 Department of Medical Sciences, University of Turin/Città della Salute e della Scienza, Via Santena 7, 10126 Turin, Italy; luca.bertero@unito.it (L.B.); paola.cassoni@unito.it (P.C.)

**Keywords:** NG2/CSPG4, CNS, vessels, gliomagenesis, development

## Abstract

Neuron glial antigen 2 (NG2) is a chondroitin sulphate proteoglycan 4 (CSPG4) that occurs in developing and adult central nervous systems (CNSs) as a marker of oligodendrocyte precursor cells (OPCs) together with platelet-derived growth factor receptor α (PDGFRα). It behaves variably in different pathological conditions, and is possibly involved in the origin and progression of human gliomas. In the latter, NG2/CSPG4 induces cell proliferation and migration, is highly expressed in pericytes, and plays a role in neoangiogenesis. NG2/CSPG4 expression has been demonstrated in oligodendrogliomas, astrocytomas, and glioblastomas (GB), and it correlates with malignancy. In rat tumors transplacentally induced by *N*-ethyl-*N*-nitrosourea (ENU), NG2/CSPG4 expression correlates with PDGFRα, Olig2, Sox10, and Nkx2.2, and with new vessel formation. In this review, we attempt to summarize the normal and pathogenic functions of NG2/CSPG4, as well as its potential as a therapeutic target.

## 1. Introduction

Oligodendrocyte-type 2 astrocyte (O2A) progenitors generate oligodendrocytes or type 2 astrocytes in cultures of perinatal optic nerves [1]. They express the neuron glial antigen 2 (NG2) or chondroitin sulphate proteoglycan 4 (CSPG4) protein, develop in the ventricular germinal zone of embryos, and proliferate and migrate to the central nervous system (CNS) after birth. They associate with axons as myelinating oligodendrocytes. O2A progenitors occur also in adults, involved in the repair of demyelinating damage [2]. NG2/CSPG4 cells expressing platelet-derived growth factor receptor α (PDGFRα) have been found to represent, in mature brain, 5% of the cells [3], and were regarded as a novel “fifth neural cell type” after neurons, oligodendrocytes, astrocytes, and microglia [3,4,5]. They became better known as “oligodendrocyte precursor cells” (OPCs). 

The so-called NG2-glia participate in neuronal functions; after focal demyelination in mice *corpus callosum*, demyelinated axons form functional glutamatergic synapses onto adult-born NG2+ OPCs migrating from the subventricular zone (SVZ) and, within the oligodendrocyte lineage, they monitor the firing patterns of surrounding neurons [6]. Synapses with neurons occur [7], and physiology and plasticity of the functional role of synapses on NG2-glia has been widely discussed [8]. 

The majority of adult NG2-glia are located in the white matter of the cerebral cortex and differentiate mostly into mature, myelinating oligodendrocytes, and gray matter. NG2-glia generate fewer mature oligodendrocytes [9,10,11]. NG2-glia are cells with a highly proliferative capacity [12,13,14] and it is, nowadays, broadly accepted that they generate oligodendrocytes, and that there is a regional heterogeneity, such that problems remain open as far as the lineage potential of NG2-glia is concerned [8].

NG2-glia represent the major population of endogenous/resident progenitor cells, capable of “reacting” to any type of injury and with the potential to repopulate areas of lesions [8]. They respond to injuries with a rapid proliferation and scar formation, but also contribute to the failure of axon regeneration [15]. They can also produce astrocytes [16] or remain as NG2-glia [13]. Embryonic NG2-glia, potentially producing astrocytes, migrate and differentiate into astrocytes that could, alternatively, be derived from NG2-expressing astrocytic progenitors in a short temporal and regional manner [4]. In vitro NG2-glia can differentiate into type 2 astrocytes [1,17,18] and, in specific cell culture conditions, O2A progenitors can self-renew and give rise to oligodendrocytes, and even, neurons [3,19,20]. In acute injuries, NG2-glia may react, accumulating cells with short processes [11,21]. Our knowledge on NG2 has been strongly supported by experiments on conventional transgenic NG2/CSPG4 knockout mice [22] and more information came from immunotoxic approaches [23]. In extraneural tissues, NG2/CSPG4 expression is important for pericyte localization to endothelial layer and interaction with endothelial cells [24]. 

NG2/CSPG4 plays an essential role in cell proliferation, migration, and metastasis [25]. Aberrant expression of NG2/CSPG4 in tumors and angiogenetic vasculature was found to be associated with an aggressive disease course in several human malignancies. It is expressed on the surface of tumor cells and vascular pericytes, besides a relatively restricted distribution in healthy tissues to be proposed as an attractive candidate to target, simultaneously, both the malignant and stromal cell compartments within the tumor [26]. Based on its structure, distribution, and functions, NG2/CSPG4 has been suggested to promote tumor progression by multiple mechanisms and represents, to date, a powerful target for chimeric antigen receptor-based T-cell (CAR-T) immunotherapy of solid and hematological malignancies [27,28,29]. 

The importance of NG2/CSPG4 in gliomagenesis and in proliferation of gliomas has been repeatedly proven. A new relevance from preclinical studies using an anti-NG2/CSPG4 CAR-T therapy in glioblastoma (GB) has been recently emphasized [30,31]. 

**Figure 1:**
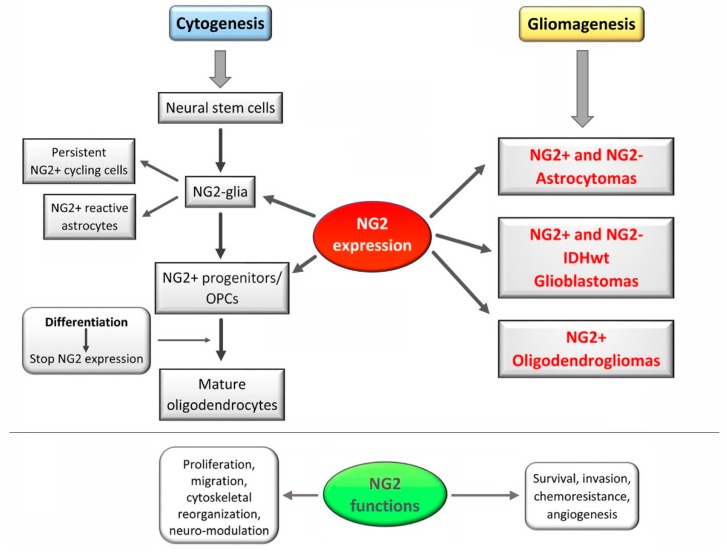
Summary scheme of NG2/CSPG4 expression during neurogenesis and gliomagenesis, and its functions in normal and pathogenetic mechanisms of the central nervous system (CNS). Expression of NG2/CSPG4 is found in subsets of normal glial cells in developing and adult CNS. It is not expressed by multipotent stem cells but is upregulated in the NG2-glia and in the partially-committed oligodendrocyte precursor cells (OPCs) that are still proliferative and motile. Upon terminal differentiation of these progenitors in mature oligodendrocytes, NG2/CSPG4 is downregulated. It is once again upregulated in pathological conditions, including malignant cancers. NG2/CSPG4 aberrant expression has been associated with gliomas where it affects cancer cell adhesion, migration, proliferation, resistance, and angiogenesis.

A summary scheme of the NG2/CSPG4 expression in the nervous cytogenesis and in gliomagenesis is illustrated in Figure 1.

## 2. NG2/CSPG4 Gene

The NG2/CSPG4 proteoglycan is encoded by the *NG2*/*CSPG4* gene, and belongs to the protein family of chondroitin sulphate proteoglycans (CSPGs). The human *NG2/CSPG4* gene is located on chromosome 15q and contains 10 exons [32]. To date, no alternatively spliced variants have been described [32].

Data on single nucleotide polymorphisms (SNPs) inside the *NG2/CSPG4* gene, extracted from the dbSNP database (NCBI, National Center for Biotechnology Information, Bethesda, USA) revealed several common polymorphisms in the *NG2/CSPG4* gene. Most of them are synonymous or missense variants, the latter affecting codons encoding different amino acids compared to the wild type template. In particular, five stop-gained SNPs have been detected in the region encoding the N-terminal portion of the protein that could cause the synthesis of a shorter protein, possibly lacking the C-terminal portion compared to the full-length one.

NG2/CSPG4 has conserved its structural and functional properties through phylogenetic evolution. Its homologue in rat and mouse shares over 80% amino acid sequence identity with the human sequence, and 90% amino acid identity with each other. Amino acid differences among the three species are spread throughout the full-length coding sequence of each protein, suggesting that their primary structure is evolutionarily conserved [33].

A 1585 base pair promoter region upstream of translation initiation site containing binding sites for p300 and CREB transcription factors regulates the *NG2/CSPG4* expression. At the post-transcription level, *NG2/CSPG4* mRNA is regulated by microRNA (miR-129-2) that binds 3’-UTR of *NG2/CSPG4* mRNA [34].

## 3. NG2/CSPG4 Structural and Functional Features

NG2/CSPG4 was first characterized as a high-molecular-weight type 1 membrane proteoglycan in rat in 1981 [35], and then identified with a mouse monoclonal antibody (mAb) on human melanoma cells [36]. 

**Figure 2:**
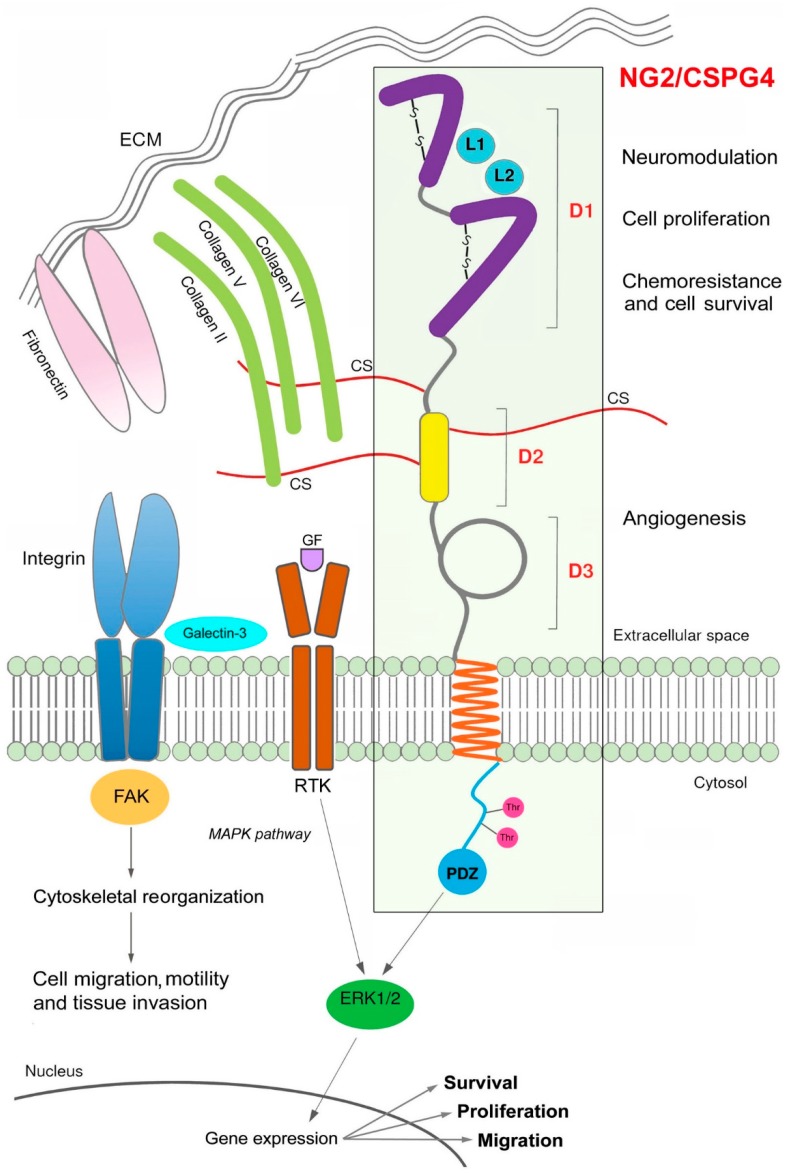
Structure and functions of chondroitin sulfate proteoglycan 4 (CSPG4). NG2/CSPG4 is a type 1 transmembrane protein composed of (1) an extensive 2225-residue N-terminal ectodomain (amino acids 1–2221), (2) a small 25-residue transmembrane domain (amino acids 2222–2246), and (3) a short 76-residue C-terminal cytoplasmic domain (amino acids 2247–2322). The extracellular ectodomain can be further divided into three subdomains: domain 1 (D1), domain 2 (D2), and domain 3 (D3). D1 is a N-terminal globular domain (amino acids 1–640) stabilized by intramolecular disulfide bonds and containing two laminin G-type motifs (L1 and L2) involved in the ligand binding at the extracellular matrix (ECM). D2 is a central large domain (amino acids 641–1590), containing 15 CSPG repeats that are the attachment sites for the chondroitin sulfate chains, collagens II, V and VI. D2 interacts with integrins and ECM proteins, and binds and presents growth factors to receptor tyrosine kinases. D3 is a globular juxtamembrane domain (D3, amino acids 1591–2221) containing N-linked oligosaccharides that bind galectin-3 and α3β1 integrin, and putative protease cleavage sites of NG2/CSPG4, leading to its shedding from the cell surface. The cytoplasmic tail, rich in proline and threonine residues, interacts with different proteins and functions as a phosphoacceptor site for the extracellular signal-regulated kinase 1/2 (ERK1/2), respectively. The PDZ domain is implicated in protein scaffolding functions. NG2/CSPG4 is, thus, implicated in cellular signaling pathways, including the mitogen-activated protein kinase pathway, through the receptor tyrosine kinase-ERK1/2 axis and the focal adhesion kinase (FAK) pathway, through the ECM–fibronectin–integrin axis. All may promote migration, proliferation, survival, and cytoskeletal reorganization, resulting in enhanced motility, invasiveness, and angiogenesis.

NG2/CSPG4 shows structural features that make it unique among members of the proteoglycan family. While most proteoglycans can be grouped into families according to structural similarities, NG2/CSPG4 does not contain structural motifs common to any of these groups [37]. It consists of a N-linked glycoprotein of 290 kDa and a proteoglycan component of about 450 kDa. This polypeptide contains several glycosylation sites and three putative glycosaminoglycan (GAG) attachment sites [37,38]. Since NG2/CSPG4 can be expressed on the cell surface both with N-linked chondroitin sulphate (CS) chain and without any GAG chain, it can be regarded as a “part-time proteoglycan” [32]. It consists of a large extracellular domain with 2,225 amino acids accounting for 95% of the protein, a transmembrane domain with 25 amino acids, and a short cytoplasmic tail of 76 amino acids [37] (Figure 2). 

The full-length NG2/CSPG4 protein is processed by sequential cleavage by the α-secretase ADAM10 and the γ-secretase complex, both expressed by OPCs, into four major fragments that are associated with different functions. The proteolytic cleavage of the extracellular domain generates a soluble 290 kDa NG2/CSPG4 ectodomain, which can be released from the cell into the extracellular matrix (ECM), and a membrane-bound C-terminal fragment (CTF, 12 kDa). The latter can be further processed by the γ-secretase complex with release of an intracellular domain (ICD, 8.5 kDa) containing the PDZ domain [39]. The proteolytic shedding results are greatly enhanced in several types of injuries [40], i.e., spinal cord injuries, multiple sclerosis, and tumors. 

Due to its structure, NG2/CSPG4 is involved in a wide range of molecular interplays, including neuromodulation, cell proliferation, migration, adhesion, and metastasis (Figure 2). 

The extended extracellular domain, still partially unknown, acts in neuronal network regulation [39] with neuromodulatory properties [41], or in endothelial cell and pericyte-related interactions [42]. Ablation of NG2-glia cells in NG2-HSVtk transgenic rats produces defects in hippocampal neurons due to neuroinflammation through the IL-1β pro-inflammatory pathway, showing that NG2/CSPG4 controls neuroimmunological functions [43]. Moreover, it has been shown in NG2/CSPG4 knockout mice that loss of NG2/CSPG4 may hamper, besides the neuronal network, the cytokine production by neural and immune cells [43].

The intracellular domain is in relation with extracellular signal-regulated kinase (ERK) and protein kinase C-alpha (PKCα). Both intervene in the regulation of proliferation, migration, invasion, cytoskeletal reorganization, survival, chemoresistance, and modulation of the neuronal network [44]. The mechanism at the basis of these functions is the NG2-dependent activation of β1 integrins, both when the two molecules are expressed in the same cells and in two different cells [45]. Enhanced proliferation is accomplished through fibroblast growth factor/fibroblast growth factor receptor (FGF/FGFR) signaling via Ras and ERK, enhanced motility through focal adhesion kinase (FAK) and enhanced survival through PI3K/Akt signaling [45]. 

The cytoplasmic domain of NG2/CSPG4 contains several structural features that are critical for its function. PDZ-type adaptor proteins mediate the interactions between NG2/CSPG4 and the actin cytoskeleton [46]; two threonine residues, which undergo differential phosphorylation by PKCα (Thr^2256^) and ERK (Thr^2314^) signaling, are implicated in cell proliferation and migration [44]. Finally, a proline-rich segment in the C-terminal half of the cytoplasmic domain may facilitate additional protein–protein interactions.

A soluble NG2/CSPG4 fragment released from tumor cells or tumor-associated pericytes can stimulate endothelial cell migration in the tumor microenvironment by interacting with galectin-3 and α3β1 integrin on the endothelial surface [47]. 

## 4. NG2/CSPG4 Expression Pattern

In terms of tissue distribution, NG2/CSPG4 is highly expressed in more than ten different adult tissues or organs, including brain, gastrointestinal tract, and endocrine organs, with poor correlation between transcript and protein levels in most of them [48]. 

Although expressed in >50 cell types, including chondroblasts, osteoblasts, keratinocytes, smooth muscle cells, and macrophages [49,50], NG2/CSPG4 expression seems to be confined to precursor or progenitor cells of epithelial and mesenchymal origin [48]. In particular, NG2/CSPG4 is not expressed by multipotent stem cells, but is upregulated when a stem cell becomes initially committed to a particular cell lineage. It would be intensely expressed by partially committed progenitors, that are still proliferative, motile, and with a retained degree of developmental plasticity, until their terminal differentiation, when NG2/CSPG4 is downregulated. Due to its widespread distribution in human tissues, NG2/CSPG4 may be regarded as a marker of an “activated” (as opposite to quiescent) status of the cells, featured by cell proliferation and motility [51].

## 5. NG2/CSPG4 in the CNS Biology

In CNS, NG2/CSPG4 has been supposed to be involved in the PDGF signaling in OPCs, acting as co-receptor of PDGFRα [52]; its expression is widely used a marker of this cell type [53]. 

NG2/CSPG4 would not be expressed by multipotent neural stem cells in primary and secondary germinal zones of the CNS, but it would be upregulated in progenitors that originate from these germinal zones, and are committed to the oligodendroglial lineage [51] (Figure 1). Through a program of proliferation and migration, oligodendrocyte progenitors populate the entire CNS and differentiate into myelinating oligodendrocytes. Their maturation is marked by downregulation of both NG2/CSPG4 and PDGFRα. In the grey matter, NG2/CSPG4+ precursors can differentiate into astrocytes.

Post-natal oligodendrocytes derive from OPCs that represent the main population of cycling cells in the adult rat brain [3]. They can be found in the SVZ, and in the gray and white matter of adults [16,20], and NG2/CSPG4 expression can be demonstrated, together with PDGFRα, in tissues fixed for <48 h in paraformaldehyde [5]. In others’ experience, NG2/CSPG4+ cells in the gray and white matter of normal brain are demonstrable only after prolonged incubations and increased antibody concentrations in comparison with glioma tissue [54].

Since a large number of cycling NG2/CSPG4+ and PDGFRα+ cells persist in the adult rodent brain and spinal cord, the existence of a third class of differentiated macroglia, designated as polydendrocytes [4] or neuroglial cells [55] of unknown functions, distinct from oligodendrocytes and astrocytes, has been discussed. Adult oligodendrocyte progenitors serve as a source of new oligodendrocytes for remyelination of demyelinated axons [56], and they proliferate in response to a wide variety of injuries to the CNS [40]. Adult polydendrocytes have morphologies distinct from those of simple neonatal progenitors [4], exhibit intimate spatial relationships with synaptic structures [57] and nodes of Ranvier [58], and receive functional synaptic input from glutamatergic neurons [59]. 

In the developing rat CNS, the co-expression of NG2/CSPG4 and PDGFRα in progenitor cells of the O2A lineage in white and gray matter starts from embryonic day 15 (E15) for PDGFRα and E17 for NG2/CSPG4, reaching the peak in the first post-natal week, and then it declines, even remaining demonstrable in adults [52]. NG2/CSPG4+ cells are located in the SVZ of mice as amplified transit C cells, and proliferate and express epidermal growth factor receptor (EGFR) and Olig2 [60,61]. When grafted, they generate hippocampal GABAergic interneurons and, therefore, represent a cell reservoir for renewal of interneurons [60]. Besides PDGFRα [62] and A2B5 [63], OPCs belatedly express 2′,3′-cyclic nucleotide phosphodiesterase (CNPase) [64] and are widespread in the adult brain [3,64], where they represent the major dividing cell population giving rise to oligodendrocytes. In the developing murine brain, NG2/CSPG4+ cells arise in three regional waves at different times [8], of which only the last one survives and expands. In adult rat brain, NG2-glia is distributed through all brain regions, including the *corpus callosum* and in gray matter [57]. 

## 6. NG2 /CSPG4 in Gliomas

NG2/CSPG4+ OPCs have been described in the development of adult gliomas [54,65]; their proliferative ability makes them a susceptible target to oncogenic transformation. This is indirectly supported by the alteration of PDGFRα signaling pathway in gliomas, the same pathway that is involved in normal development of oligodendrocytes by controlling proliferation and migration of OPCs [66]. 

Experimentally, in a Ctv mouse model, tumor induction was demonstrated to be restricted to CNPase-expressing OPCs, and PDGFβ transfer was proven to induce gliomas in 33% of cases [67]. NG2/CSPG4 expression has been found to be widespread in murine gliomas [68,69,70,71]. In rat gliomas, transplacentally induced by *N*-ethyl-*N*-nitrosourea (ENU), NG2/CSPG4 was found to be diffuse in oligodendrogliomas, where cells failed to differentiate into mature oligodendrocytes. In slowly growing gliomas, cells expressed NG2/CSPG4, as well as Olig2, Sox10, and Nkx2.2, all markers of committed progenitor cells to the oligodendroglial lineage, but not O4, a marker of late and adult OPCs [72]. 

Before the discovery of NG2/CSPG4, CS could be biochemically and histochemically demonstrated in gliomas, together with GAGs. They were found to be associated with vessel walls and cytoplasmic membranes of tumor cells, in an inverse relationship with dedifferentiation [73,74]. They were also variably distributed, especially in regressive events [75,76], and they showed a particular behavior in ENU transplacentally induced rat brain tumors [77,78,79]. In particular, Alcian blue positivity for CS was found in isomorphic ENU oligodendrogliomas, and only in the peripheral part of polymorphic gliomas [77], in agreement with recent NG2/CSPG4 findings [72]. The latter authors, very interestingly, could not obtain neurospheres (NSs) from low-grade tumors, but they observed that after irradiation, surviving cells were NG2/CSPG4+; the stem cell hypothesis of ENU gliomas was, therefore, considered to be very unlikely. However, murine oligodendroglioma cells show characteristics of OPCs [80]. 

PDGFRs have been found to be overexpressed in human malignant astrocytomas [81]; moreover, expression of NG2/CSPG4 and PDGFRα was identified in oligodendrogliomas, pilocytic astrocytomas and, heterogeneously, in GBs [54,82]. Neoplastic cells showed, predominantly, cell surface staining with anti-NG2/CSPG4 and -PDGFRα antibodies, with a distinct morphology compared with that of resident NG2/CSPG4+ cells in normal brain [54]. PDGFR-AA high expression levels and gene mutations were regarded as a feature of the proneural subtype of GBs [83]. Diffuse gliomas expressed NG2/CSPG4, PDGFRα, and Olig2, that are characteristic of OPCs [84,85,86]. In summary, NG2/CSPG4 expression can be considered as variable in human gliomas [51] (Figure 1). As a matter of fact, even though it correlates with the malignancy grade [54,84,87], no pattern of co-expression among NG2/CSPG4, Olig2, and PDGFRα has, until now, been found in GB, so that it is not known whether the pattern found in the CNS is recapitulated in GB [69]. In the latter, NG2/CSPG4 expression occurs in 67% of the cases and in NS [22,79], influencing patient survival [31,84,88].

In ninety-six studied GBs, 50% showed high expression level of NG2/CSPG4 in tumor cells and vessels, together with Nestin and Vimentin, but not with CD133. NG2/CSPG4+ tumor cells revealed upregulation of peroxiredoxin-1 (PRDX-1), and were resistant to ionizing radiation; the knockdown of PRDX-1 slowed cell growth and sensitized to radiation. NG2/CSPG4 could be, therefore, an important prognostic factor [89].

In the CNS, regardless of pericytes, NG2/CSPG4 is also a marker of activation status [51] and, since NG2/CSPG4+ cells are the most important population of cycling cells in the adult CNS [3,90], gene mutations can accumulate, leading to the genesis of gliomas [51,91]. NG2/CSPG4 distribution does not correspond to that of Olig2, that marks oligodendroglial nuclei in normal brain, and in oligodendroglial tumors and, to a lesser extent, in astrocytic tumors, being mutually exclusive with GFAP [92]. NG2/CSPG4, PDGFRα, Olig2, Sox10, and Nkx2.2 were preferentially found in human diffuse gliomas with oligodendroglioma or oligoastrocytoma morphology [93,94]. As a whole, all these observations are in line with the origin of most gliomas from the subcortical white matter rich in OPCs expressing NG2/CSPG4, PDGFRα, and Olig2 [71]. 

NG2/CSPG4+ cells in gliomas conditions poor survival as they promote cell proliferation and motility via β1 integrins and growth factors [51]. It has been shown that NG2/CSPG4 contains binding sites for FGF2 and PDGFα and, once blocked, proliferation of OPCs is inhibited [51]. Moreover, chemoresistance is promoted by NG2/CSPG4 by activating α3β1 integrin-dependent PI3K/Akt signaling, and there is an inverse relationship with apoptosis, demonstrated by its restoration after siRNA knockdown of NG2 [95]. NG2/CSPG4-dependent α3β1 integrin signaling plays a role not only in tumor progression, but also in the maturation and function of tumor blood vessels [45]. In gliomas, NG2/CSPG4 is one of the highly upregulated proteoglycans [96] that increases the invasive and migratory capabilities of glioma cells by facilitating interactions with ECM proteins, such as collagens II, V, VI, and laminin [97]. 

**Figure 3:**
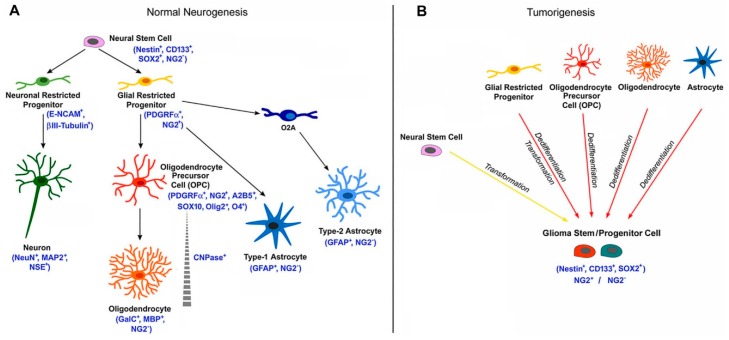
Scheme of the NG2/CSPG4 expression in the nervous cytogenesis and gliomagenesis. (**A**) Scheme representing the expression of NG2 marker during normal neurogenesis and the different cell fates of NG2-glia. Canonically, NG2-glia (NG2+ glial restricted progenitors) have the capability to proliferate and differentiate into oligodendrocyte precursor cells (OPCs) giving, then, rise to oligodendrocytes in the immature and mature brain. However, NG2-glia can also differentiate into astrocytes. Additionally, in vitro, NG2-glia can differentiate into type-2 astrocytes through the O2A progenitor cells. (**B**) NG2+ cells can also be considered as potential cells for the origin of malignant glioma. During tumorigenesis, the glioma stem cell is believed to derive from transformation of neural stem cell or from dedifferentiation and transformation of NG2+ glial restricted progenitors, OPCs, or mature cells (astrocytes and oligodendrocytes). The arising tumor cells show potential for self-renewal, and express markers associated with both stem and progenitor cell types.

A scheme representing NG2/CSPG4 expression during normal neurogenesis and the different cell fates of NG2-glia is illustrated in the Figure 3.

## 7. NG2/CSPG4 in Blood Vessel Development

Another important function of NG2/CSPG4 is its role in blood vessel development and its expression in pericytes involved in tumor progression [98]. It has been also demonstrated in endothelial cells of normal brain vessels [99], as well in the proliferated tumor vessels of malignant gliomas [54,87]. NG2/CSPG4 is, therefore, expressed in vascular mural cells [100]. Tube formation without endothelial cells, but with NG2/CSPG4+ and PDGFRα+ cells, can also occur [101]. In the developing human brain, endothelial cells are preceded and even guided by migrating pericytes during organization of the growing vessel wall [102], as if pericytic NG2/CSPG4 could mediate endothelial cell recruitment [47]. 

A differential NG2/CSPG4 expression from pericyte subsets has been recently described [103]. In the human fetal cortex, poorly stabilized vascular structures contain NG2/CSPG4 expressing pericytes that are responsible for neoformed vessels [102]. Conversely, NG2/CSPG4 is downregulated in pericytes associated with quiescent vessels, and absent or not detectable in pericytes of stable vessels in the adult healthy human brain [102]. Notably, only a specific type of pericyte, expressing both NG2/CSPG4 and Nestin, would be recruited during tumor angiogenesis [104].

What happens during tumor angiogenesis between endothelial cells and pericytes has been widely discussed, and aberrations in their relationship have been considered important for angiogenesis and metastasis [105]. Blood vessel development is altered in NG2/CSPG4-null tumors [51]. By knocking down NG2/CSPG4 in pericytes by siRNA transfection, there is a 60% reduction of β1 integrin activation and 40% of FAK phosphorylation with a concomitant decrease of pericyte proliferation and migration [45]. It is possible that the NG2/CSPG4 ectodomain shed from pericytes, after proteolytic cleavage, recruits, at a distance, endothelial cells to sites of angiogenesis, and that it activates β1 integrin in endothelial cells. The same mechanisms can occur in the recruitment of macrophages to the tumor [45]. 

It has been shown that GB stem cells (GSCs) generate the majority of vascular pericytes and that the selective elimination of GSC-derived pericytes disrupt the neovasculature and inhibit tumor growth. Most pericytes in tumor are derived from neoplastic cells. GSCs are recruited toward endothelial cells via the SDF-1/CXCR4 axis, and become pericytes by transforming growth factor β (TGF-β). Thus, GSCs contribute to vascular pericytes that may actively remodel perivascular niches. They can be, therefore, a therapeutic target [106]. In the neoangiogenesis of GB, vascular pericytes begin to increase together with the disruption of the brain–blood barrier, and become a good marker of neovascularization [107]. 

**Figure 4:**
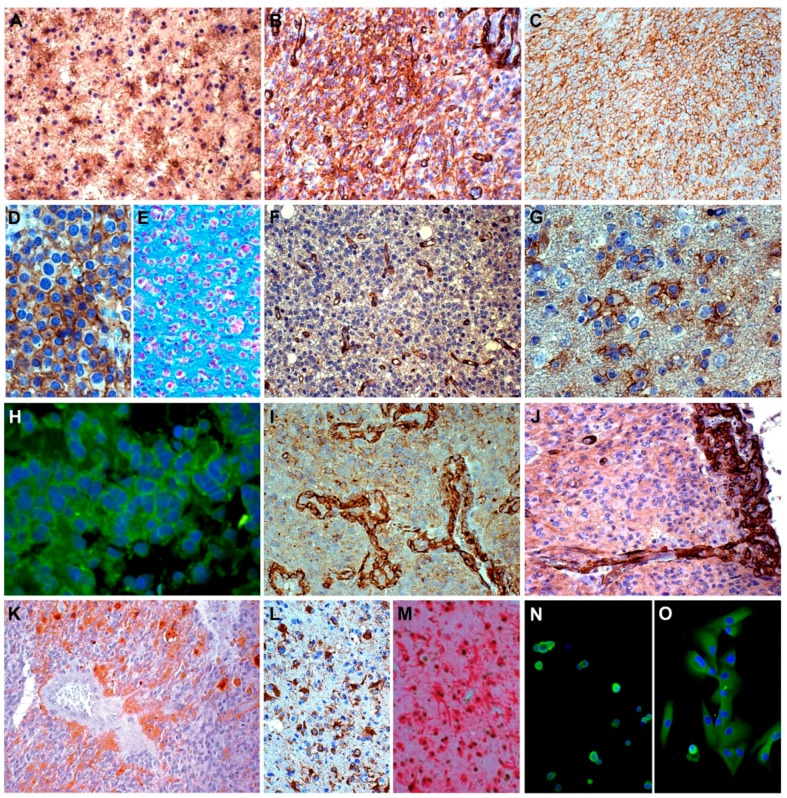
NG2/CSPG4 immunohistochemistry. (**A**) World Health Organization (WHO) grade II astrocytoma. Negative tumor cells and NG2/CSPG4-positive reactive astrocytes; DAB, 400×. (**B**) IDH-wild type glioblastoma (GB). NG2/CSPG4-positive area; DAB, 200×. (**C**) *Id.* Diffuse NG2/CSPG4 staining on cell membranes; DAB, 200×. (**D**) WHO grade III oligodendroglioma. NG2/CSPG4-positive area with honeycomb appearance; DAB, 400×. (**E**) *Id.* Alcian blue staining; 200×. (**F**) *Id.* NG2/CSPG4-negative tumor cells and -positive endothelial cells; DAB, 200×. (**G**) *Id.* Isolated NG2/CSPG4-positive tumor cells in infiltration area; DAB, 400×. (**H**) *Id.* NG2/CSPG4 expression in tumor cells; green immunofluorescence (IF), 400×. (**I**) IDH-wild type GB. Strong NG2/CSPG4 expression in vascular pericytes; DAB, 200×. (**J**) *Id.* NG2/CSPG4-positive vascular pericytes in glomerulus with sprouting; DAB, 200×. (**K**) *Id.* Negative tumor cells and NG2/CSPG4-positive reactive astrocytes in infiltration; DAB, 200×. (**L**) *Id.* NG2/CSPG4-positive reactive astrocytes; DAB, 200×. (**M**) *Id.* ATRX+/GFAP+ reactive astrocytes, double staining; DAB/Fast Red, respectively, 200×. (**N**) *Id.* GB-derived cell lines, neurospheres. Most cells are variably positive for NG2/CSPG4, but some are negative; green IF, 200×. (**O**) GB-derived cell lines, adherent cells are weakly positive for NG2/CSPG4; green IF, 200×.

Personal findings on the NG2/CSPG4 protein expression in human gliomas and GB-derived cell lines are presented in the Figure 4.

## 8. NG2/CSPG4 in the Treatment of Gliomas

NG2/CSPG4 is involved in cell proliferation through FGF2 and PDGFRα, and 80% of GBs express it together with PDGFRα and Olig2, but not as a normal brain pattern [51]. However, most of proliferating cells are NG2/CSPG4+, and half of NG2/CSPG4+ cells proliferate. Transplants of GB-derived NG2/CSPG4+ human cell lines in mice, subcutaneously in the hind limbs and orthotopically in the forebrain, compared with NG2/CSPG4+ cells from the same tumor, overexpress genes associated with aggressive tumorigenicity, including mitosis and cell cycling module genes (*MELK, CDC, MCM, E2F*) correlating with poor survival in GB [108]. This confirms NG2/CSPG4 could be a target of therapies and not only for tumors, including gliomas [33]. The possibility to exploit the theranostic properties of NG2 has been greatly emphasized [33,48]. 

High NG2/CSPG4 expression positively correlates with multidrug resistance mediated by increased activation of α3β1 integrin, PI3K/Akt signaling, and their downstream targets, promoting cell survival [89,95]. It was demonstrated that NG2/CSPG4 knockdown with shRNAs incorporated into lentiviral vectors attenuated β1 integrin signaling, revealing potent antitumor effects and further sensitized tumor cells to cytotoxic treatment, in vitro and in vivo [95]. NG2/CSPG4 may represent an effective therapeutic target in several cancer subtypes. In intracranial melanomas, the NG2/CSPG4 ablation by siRNA produced a lesser efficient vasculature in the tumor [42]. 

A reduction of growth was already demonstrated in xenografts of the human glioma cell line U87-MG in athymic nude mice, obtained by chemoimmunoconjugates of the glioma-reactive anti-NG2/CSPG4 mAb 9.2.27 and vinblastine [109]. Similarly, in xenografts of GB-derived cell lines overexpressing NG2/CSPG4, the abrogation of its function by intracerebral delivery of lentivirally encoded shRNAs reduced tumor growth and angiogenesis [26]. 

Targeting NG2/CSPG4 with mAb 9.2.27 and activated natural killer cells inhibited the tumor growth and improved the survival of GB-bearing animals with the establishment of a pro-inflammatory microenvironment [110,111]. Similar effects were obtained by miR-129-2 [34]. GB cell viability was significantly reduced by ablating NG2/CSPG4 and GD3(A), a ganglioside expressed by developing migratory glia, using a Mab-Zap saporin immunotoxin system, compared to single epitope targeting [112]. In a rat model of GB, the combination of NK cells and mAb 9.2.27 led to growth reduction, detected by contrast enhanced magnetic resonance imaging [113]. Using the Cre-lox method for cell type-specific ablation of NG2/CSPG4 [42,114], the vascularization of tumors resulted in impaired intracranial implantations of B16F10 melanoma cells in mice, via loss of NG2/CSPG4-mediated activation of β1 integrin signaling in pericytes [115].

Due to the almost exclusive expression from tumor cells, NG2/CSPG4 is an attractive candidate for antibody-based approaches, including specific anti-NG2/CSPG4 antibodies and immuno-based therapies, in particular, for CAR-T immunotherapy of solid tumors [29,115,116]. Anti-NG2/CSPG4 mAbs have been shown to inhibit tumor progression by blocking ligand access to the NG2/CSPG4 extracellular binding sites. Therefore, NG2/CSPG4-directed antibody conjugates get selectively internalized by NG2/CSPG4-expressing tumor cells by endocytosis [116]. Due to selective NG2/CSPG4 upregulation from tumor-associated pericytes, this approach may also contribute to tumor regression via inhibition of neoangiogenesis [117,118].

The development of immunotherapy provided significant progress in the treatment of indolent and metastatic tumors, including the development of genetic engineering technologies that redirect T lymphocytes to recognize and target a wide variety of tumor antigens. T cells are activated with redirected specificity via expression of CAR-Ts. CARs are hybrid proteins in which the binding moiety, derived from a monoclonal antibody, is fused with a signaling molecule of the CD3/T cell receptor complex and co-stimulatory endodomains. In order to overcome the necessity for T cells to recognize tumor antigens presented by the major histocompatibility complex (MHC), CAR-T cells are genetically modified to express a chimeric T-cell receptor that recognize the antigen of interest and redirect cytotoxic T cells toward tumor cells. Upon insertion in T cells, CARs confer MHC-independent cytotoxic activity to T cells and promote T-cell proliferation, activation, and persistence both in vivo and in vitro [30]. Clinical trials of CAR-transduced peripheral blood lymphocytes have been previously shown to cause remission of both solid and hematological human malignancies. In particular, it has been shown that redirected T cells expressing a NG2/CSPG4-specific CAR could represent a potential strategy to target a broad spectrum of indolent solid tumors [25,118]. 

Due to the established capability to block the progression of several solid tumors, NG2/CSPG4 has been chosen as novel target for CAR-T cell therapies in GB. The anti-NG2/CSPG4 CAR-T seems to overcome both tumor escape and heterogeneity of other tumor-associated antigens analyzed in previous clinical studies as CAR-T cell putative candidates for malignant gliomas. Anti-NG2/CSPG4 CAR-transduced T cells recognize and kill GB stem cells [26,118]. A recent preclinical study showed that anti-NG2/CSPG4 CAR-T cells can successfully induce growth arrest in GB-derived NS and in glioma xenograft models, without any signs of immune evasion [31]. NG2/CSPG4 was highly expressed in 31 of 46 (67%) GB tumor specimens, in lower amounts in 15 of 46 (33%) and associated with a shorter survival. Notably, the anti-NG2/CSPG4 CAR-T therapy was also effective in GB-derived NS expressing moderate to low NG2/CSPG4 levels. This effect was mediated by the in vivo upregulation of NG2/CSPG4 on tumor cells due to production of TNF-α from microglia surrounding the tumor. Antigen-activated CAR-T cells themselves produce TNF-α in the glioma tumor microenvironment [119]. The constitutive and TNF-α-inducible NG2/CSPG4 expression contributes to reduce the risk of tumor cell escape when target antigens are heterogeneously distributed on tumor cells [31].

The therapeutic strategy should keep in mind some peculiar phenotypic features of GB. One is the surrounding of circumscribed necroses by GSCs/progenitors spared by the advancing necrotic process or induced by microenvironment/necrosis [120], and the other is what emerges from a recent paper [121]. It is said that FGF1 is essential in the maintenance of stemness in GB and that heparin-binding EGF and IL-1β increase sphere forming ability. OPCs and macrophages/microglia proliferate at the tumor border, causing GB cells to acquire stem cell profiles and chemoresistance. This sanctuary is proposed to be called “border niche” and is very important for therapeutic strategies [121].

An interesting idea emerges from a study that demonstrated that, in GB cells and pericytes, ICAM-1 underlies NG2/CSPG4 expression. Silencing NG2/CSPG4 in human placenta ICAM-1 of the derived pericytes increases, mediated by ERK1/2. In cultures of A1207 GB cell lines, downregulation of NG2/CSPG4 increases ICAM-1 expression. The resulting increase of ICAM-1 on the cell surface promotes leukocyte binding, and this could be a target for immune response regulation [122].

## 9. Conclusions

In the last decades NG2/CSPG4 was demonstrated to be a key player in CNS development, in neuronal function, and in experimental and human glial tumors. Its participation in CNS development, angiogenesis, and gliomagenesis emphasizes its role as a target of therapeutic attempts. Experimentally, important results have been achieved, and it is now expected that in therapy of human gliomas its instrumental use will contribute to the defeat of glial neoplasia.

The study of NG2/CSPG4 proved to be very useful in further comprehension of CNS biology, especially for its involvement in the nervous cytogenesis concerning either neurons or glia cells, and in normal angiogenesis. In gliomas, it appears to be important in establishing their origin, to ameliorate prognostic possibilities and as a potential therapeutic target with CAR-T therapy. The dynamic expression of NG2/CSPG4 during cytogenesis could be exploited to establish the beginning of neoplastic transformation in gliomas and its significance in each molecular subtype. It would be interesting to verify possible correlations with stemness antigens, such as Nestin, Sox2, CD133, or differentiation antigens, such as GFAP, galactocerebroside C, and βIII-tubulin. 

In human gliomas, there are too few studies concerning NG2/CSPG4 correlation with survival and with chemo- and radiotherapy. Concerning its use as a therapeutic target, it should be taken into account its heterogeneity in gliomas, and that its knockdown by systemic administration would disturb normal cytogenesis and angiogenesis. On the other hand, its intratumor local administration, even though less harmful, is still technically uncertain. Studies are in progress to improve this possibility. Immunotherapy approaches using CAR-Ts could be an effective treatment modality in GB that overcomes tumor escape and NG2/CSPG4 intratumor heterogeneity. 

It has been emphasized that ablation of NG2-glia produces “deficits in excitatory glutamatergic neurotransmission and astrocytic extracellular glutamate uptake and induces depressive-like behaviors in mice” and this is through FGF2 [123]. This casts some doubts on the possibility to employ anti-NG2/CSPG4 antibodies in the therapy of gliomas or, at least, via a systemic administration. This point would necessitate further investigation.

## Abbreviations

CAR-T	chimeric antigen receptor-based T cell
CNPase	2′,3′-cyclic nucleotide phosphodiesterase
CNS	central nervous system
CS	chondroitin sulphate
CSPG4	chondroitin sulphate proteoglycan 4
EGFR	epidermal growth factor receptor
ENU	*N*-ethyl-*N*-nitrosourea
ERK	extracellular signal-regulated kinase
FAK	focal adhesion kinase
FGF	fibroblast growth factor
GAG	glycosaminoglycan
GFAP	glial fibrillary acidic protein
GB	glioblastoma
GSC	glioblastoma stem cell
NG2	neuron glial antigen 2
Nkx2.2	homeobox protein Nkx2.2
O2A	oligodendrocyte-type 2 astrocyte
OLIG2	oligodendrocyte lineage transcription factor 2
OPC	oligodendroglial precursor cell
PDGFR	platelet-derived growth factor receptor
PKCα	protein kinase C-alpha
SOX10	SRY-related HMG-box 10
SVZ	subventricular zone
